# A tip-tilt-piston electrothermal micromirror array with integrated position sensors

**DOI:** 10.1038/s41378-024-00835-w

**Published:** 2025-03-07

**Authors:** Anrun Ren, Yingtao Ding, Hengzhang Yang, Qiangqiang Liu, Teng Pan, Ziyue Zhang, Huikai Xie

**Affiliations:** 1 School of Integrated Circuits and Electronics, Beijing Institute of Technology, 100081 Beijing, China; 2 Engineering Research Center of Integrated Acousto-opto-electronic Microsystems, Ministry of Education of China, 100081 Beijing, China; 3 Chongqing Institute of Microelectronics and Microsystems, Beijing Institute of Technology, 400030 Chongqing, China

**Keywords:** Micro-optics, NEMS, Sensors

## Abstract

A tip-tilt-piston 3 × 3 electrothermal micromirror array (MMA) integrated with temperature field-based position sensors is designed and fabricated in this work. The size of the individual octagonal mirror plates is as large as 1.6 mm × 1.6 mm. Thermal isolation structures are embedded to reduce the thermal coupling among the micromirror units. Results show that each micromirror unit has a piston scan range of 218 μm and a tip-tilt optical scan angle of 21° at only 5 V_dc_. The micromirrors also exhibit good dynamic performance with a rise time of 51.2 ms and a fall time of 53.6 ms. Moreover, the on-chip position sensors are proven to be capable for covering the full-range movement of the mirror plate, with the measured sensitivities of 1.5 mV/μm and 8.8 mV/° in piston sensing and tip-tilt sensing, respectively. Furthermore, the thermal crosstalk in an operating MMA has been experimentally studied. The measured results are promising thanks to the embedded thermal isolation structures.

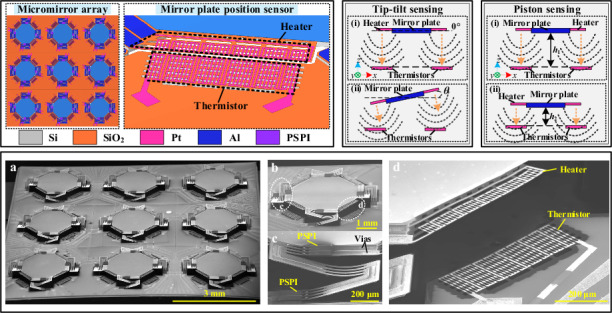

## Introduction

Microelectromechanical system (MEMS) micromirror arrays (MMAs) have been widely applied in various fields, such as optical phased arrays (OPAs)^1–3^, displays^4,5^, adaptive optics systems^6^, optical communication networks^7,8^, and optical cross-connects (OXCs)^9^. The essential part of a micromirror is a microactuator. There are mainly four actuation mechanisms for MMAs, i.e., electrothermal^10,11^, electromagnetic^12,13^, electrostatic^14^, and piezoelectric^15^. Among them, electrothermal actuation enabled by electrothermal bimorph beams shows advantages of large actuation range, low driving voltage, and high fill factor. However, the fluctuations of the environmental conditions and the fatigue and creep of the thin films in the bimorph structures can cause responsivity variations of such electrothermal micromirrors. This issue will be further amplified in electrothermal MMAs due to the increased non-uniformity among the micromirror units. Therefore, it is crucial to have a means to monitor and control the position (piston displacement and tip-tilt angle) of the mirror plate of each micromirror unit in real time.

For electrothermal MMAs, their displacement ranges are large (typically hundreds of microns) and their fill factors are high. Capacitive sensing^16–18^ is difficult to handle large displacement, while piezoresistive sensing^19–21^ runs into serious issues with the relatively high temperature in the electrothermal bimorph beams. Inductive eddy current sensing^22–24^ and optical sensing^25–27^ both require a dedicated assembly of extra components, which is difficult to be implemented in MMAs with the fill factor as an important parameter. Recently, we proposed a novel position sensing method based on thermal convection^28^, which can effectively detect the position of a single electrothermal micromirror. However, its feasibility in the MMA with a high fill factor still needs investigation considering the possible thermal crosstalk among different sensors.

In this work, this temperature field-based position sensing method is applied to a 3 × 3 lateral-shift-free (LSF) electrothermal MMA. The fill factor of the MMA is optimized by enlarging the size of the mirror plate and reducing the pitch between adjacent micromirror units. Consequently, the fill factor is increased from 12% in our previous work^29^ to 25% in this work. Note that the utilized sensing method is an on-chip solution and does not need to assemble off-chip components, which also contributes to the high fill factor. Besides, thermal isolation structures are incorporated in the micromirrors between the sensors and the mirror plate as well as the substrate, which can effectively reduce the thermal coupling in the MMA. In the following sections, the design, fabrication, and characterization of the MMA containing position sensors are described.

## Methods

### Design of the MMA

**Fig. 1:**
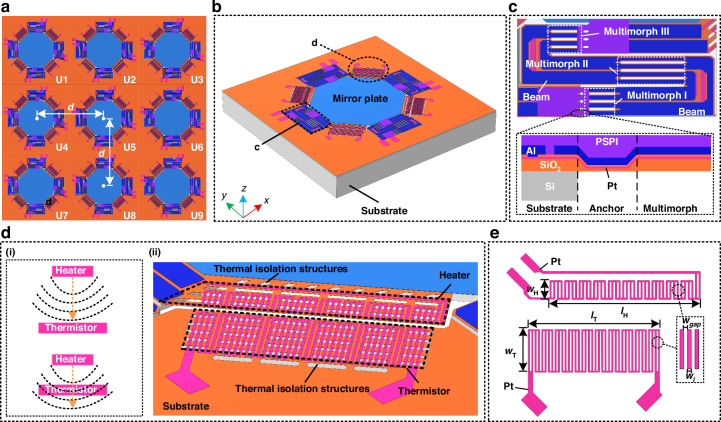
Schematic illustrations of the electrothermal MMA containing position sensors. **a** Top view of the entire MMA. **b** A single micromirror unit with position sensors. **c** Enlarged top view of an actuator consisting of two beams and three multimorphs. **d** (i) Working principle and (ii) enlarged side view of the position sensor. **e** Structural design of the position sensor

Figure 1a shows the top view of the proposed 3 × 3 electrothermal MMA, and one single micromirror unit is depicted in Fig. 1b, where the position sensors are located at the four corners of the mirror plate suspended by four LSF electrothermal bimorph actuators. As shown in Fig. 1c, each electrothermal actuator in the micromirror is composed of three multimorphs (PSPI/Al/SiO_2_) with two beams connected in between. Notably, photosensitive polyimide (PSPI) is attached to the actuator to improve the robustness of the micromirror^30^. As shown in Fig. 1d, each integrated position sensor contains a heater connected to the mirror plate and a thermistor on the substrate, which functions by sensing the variation of the temperature field produced by the heater with the thermistor. Concretely speaking, a temperature field is generated when the heater is heated, which changes along with the synchronous movement of the heater and the mirror plate and causes the resistance change of the thermistor. Detailed description of the working principle is given in Fig. S1. As indicated in Fig. 1d(ii), there are thermal isolation structures between the heater and the mirror plate to reduce heat loss and thermal coupling back to the mirror plate, improving the heating efficiency. The thermal isolation structures are also incorporated between the thermistor and the substrate to prevent heat from flowing to the substrate, which can enhance the sensitivity of the sensor. As shown in Fig. 1e, the heater and the thermistor are both designed to be grating structures to improve the contact area with surrounding air and reduce the convective thermal resistance between the heater and air, which can further improve the heating efficiency. Besides, Pt is selected as the material for the heater and the thermistor due to its high temperature coefficient of resistance (TCR). The designed structural parameters are shown in Table 1.

**Table 1:** Structural parameters of the MMA

Parameter	Value
Array footprint	9.6 mm × 9.6 mm
Size of micromirror	1.6 mm × 1.6 mm
Pitch of micromirror units (*d*)	3 mm
Length of Multimorph I/III	140 μm
Length of Multimorph II	280 μm
Length of beam	300 μm
Length of heater (*l*_H_)	450 μm
Length of thermistor (*l*_T_)	550 μm
Width of Multimorph	20 μm
Width of beam	150 μm
Width of heater (*w*_H_)	110 μm
Width of thermistor (*w*_T_)	200 μm
Width of Pt resistance	10 μm
Width of gap (*w*_*gap*_)	7 μm
Width of Pt line (*w*_*l*_)	7 μm
Thickness of Al	1 μm
Thickness of Pt	0.15 μm
Thickness of SiO_2_	1 μm
Thickness of PSPI	4 μm

Next, a finite element method (FEM) model of the MMA is built and analyzed using COMSOL software. The properties of the employed materials are listed in Table S1. The behaviors of the position sensor are studied by FEM, and the parameters used in the thermal convection simulations are listed in Table S2. The working performance of the MMA is simulated, as shown in Fig. S2, where a single micromirror can reach a piston vertical displacement of 200 μm and a tip-tilt optical scan angle of 20°. Figure S3a, b shows the simulated temperature field distributions near the thermistors when the mirror plate locates at different positions. It is shown that the temperature of the thermistor increases while the mirror plate vertically approaching the substrate, and it is higher for the thermistor closer to the heater than that further to the heater. Fig. S3d and e plot the temperature variation curves for piston sensing (Δ*T*_h_) and tip-tilt sensing (Δ*T*_θ_). Note that the theoretical sensing ranges of the sensors for piston vertical displacement and tip-tilt optical scan angle can reach 200 μm and 20°, respectively, covering the motion ranges of the micromirror unit.

In summary, the engineered tip-tilt-piston electrothermal MMA containing on-chip temperature field-based position sensors shows several advantages. First, the MMA features a high fill factor, both piston and tip-tilt motions, a large scan range, and a low driving voltage. Second, it achieves the integration of on-chip position sensors without any off-chip sensing components, and the sensing range of the sensors can cover the entire motion of the mirror plate. Third, the position of each micromirror unit can be detected separately, enabling the independent position control of multiple units. Last, the integrated sensors have a simple structure and a convenient fabrication process that is fully compatible with the micromirror.

### Device fabrication process

**Fig. 2:**
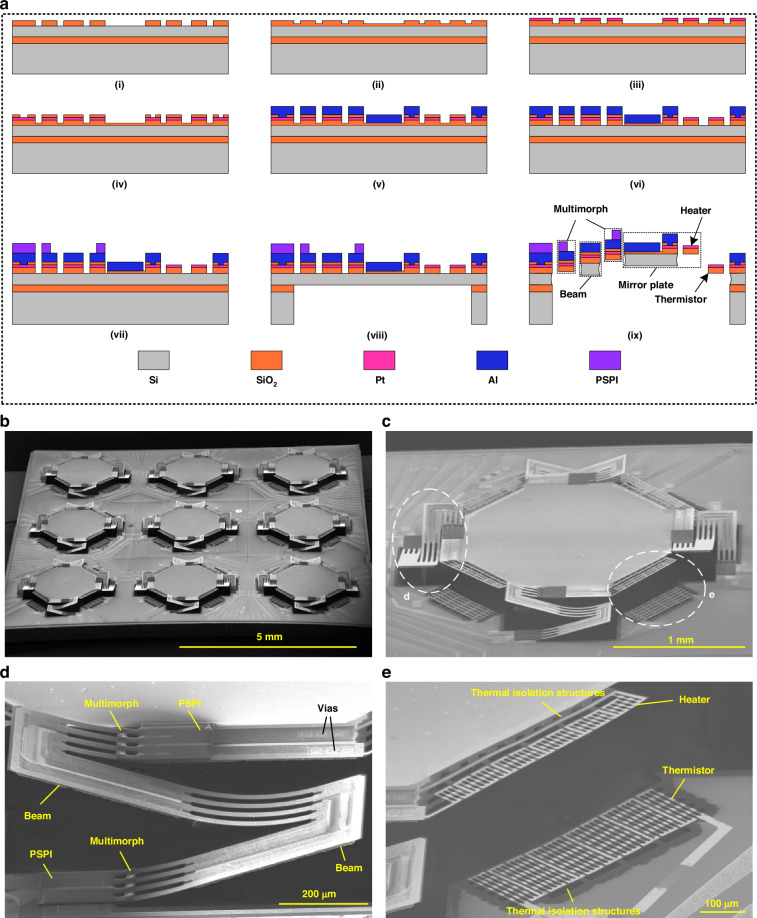
Fabrication process and corresponding results of the MMA. **a** Fabrication process. **b** SEM image of the entire MMA after release, where all micromirror units are supported by LSF electrothermal actuators. **c** Close-up view of a micromirror unit. **d** Enlarged close-up view of the actuator. **e** Enlarged close-up view of the position sensor

Figure 2a illustrates the process flow of the proposed MMA, which is realized by a simple surface- and bulk-combined MEMS process based on SOI wafer. The thicknesses of the handle layer, buried oxide (BOX) layer, and device layer are 400 μm, 1 μm, and 20 μm, respectively, where the BOX layer is used as the etch-stop layer during the back-side deep reactive ion etch (DRIE) process. The detailed fabrication steps are as follows. In Fig. 2a(i), a 1 μm-thick SiO_2_ layer is deposited by plasma-enhanced chemical vapor deposition (PECVD) at 300 °C and patterned through buffered oxide etch (BOE) process. In Fig. 2a(ii), a 0.1 μm-thick SiO_2_ layer is deposited. In Fig. 2a(iii), a 0.15-μm-thick Pt layer is sputtered and patterned through lift-off, which forms the heater and the thermistor of the mirror plate position sensor and the resistance of the actuator. In Fig. 2a(iv), another 0.1-μm-thick SiO_2_ layer is deposited as the insulation layer between the Pt layer and Al layer, followed by reactive ion etch (RIE) process to form the vias. In Fig. 2a(v), a 1 μm-thick Al layer is deposited and patterned by RIE, which forms the structure layer of the multimorph, the mirror plate, and the electrical wires distributed on the substrate. In Fig. 2a(vi), the 0.2 μm-thick SiO_2_ layer is patterned by RIE to define the shape of the multimorph. In Fig. 2a(vii), the PSPI anchor is fabricated by spin coating and patterned, accomplishing the multimorph. In Fig. 2a(viii), a Si cavity is etched until the BOX layer from the backside of the SOI wafer by DRIE, followed by the removal of the BOX layer by RIE. Finally, the micromirror is released from the frontside by isotropic etching using xenon difluoride (XeF_2_), as shown in Fig. 2a(ix).

Figure 2b shows the SEM image of the fabricated tip-tilt-piston electrothermal MMA after release. The mirror plate supported by the four actuators is elevated by about 210 ± 15 μm above the substrate due to residual stress. The elevated height can be controlled by the structural design in size, selection of material with different thermal expansion coefficients and temperature in the fabrication processes of the electrothermal multimorph actuator, which affects the quasi-static characterization and frequency response of the device. The size of each mirror plate is about 1.6 mm × 1.6 mm, and the MMA footprint is about 9.6 mm × 9.6 mm, corresponding to a fill factor of 25%. In addition, the fill-factor of the MMA can be improved by decreasing the gaps between adjacent mirror plates through minimizing the areas occupied by the supporting frames. With the design of the current mirror units, a large fill-factor of 50% can be expected. As the fill-factor is the area ratio of the mirror plate over the mirror unit, increasing the mirror plate size can also increase the fill-factor if the supporting frames are kept the same. The fill-factor of the MMA can be further improved by integrating through-silicon-via (TSV) for wiring. Figure 2d, e shows the close-up SEM images of the actuator and the position sensor, respectively, where the thermal isolation structures are clearly observed. The cantilevered heaters and thermistors are supported by SiO_2_ beams, and the underneath Si is etched.

### Device characterization results

#### Test setup

**Fig. 3:**
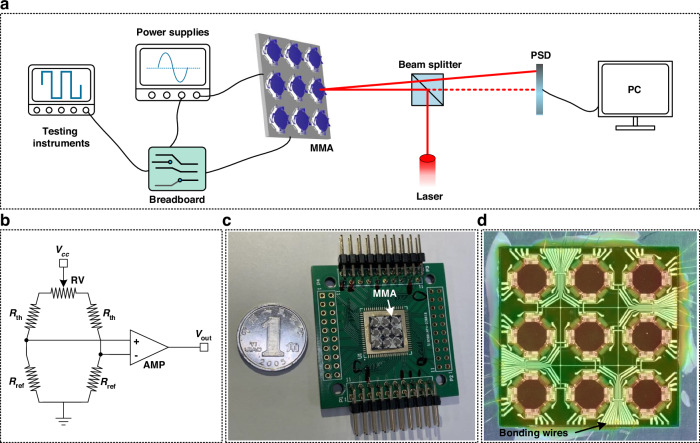
Test setup and the packaged device. **a** Test setup. **b** Diagram of the Wheatstone bridge circuit on the breadboard. **c** Packaged MMA on the PCB board. **d** Enlarged optical photo of the MMA after packaging

Figure 3 shows the schematic diagram of the measurement setup for characterization of the MMA. The power supplies include DC power supply and functional waveform generator to power the MMA and the Wheatstone bridge circuit which is used to evaluate the resistance variation of the thermistors, respectively. Besides, a position-sensitive detector (PSD) is used to calibrate the actual position of the mirror plate with the data obtained by the sensor in both the vertical displacement and the optical scan angle. The testing instruments include a spectrum analyzer, oscilloscope, and digital multimeter to acquire the output signal of the circuit.

As the TCR of thin-film Pt resistor is different from that of bulk Pt and depends on the deposition process, it is characterized within a temperature-controlled oven as shown in Fig. S5a. Figure S5b plots the measured curve of resistance versus temperature, and the TCR is calculated to be 0.00178/°C. The resistances of the Pt resistors in different mirror plate position sensors in an MMA are listed in Table S4, showing a good consistency.

#### Quasi-static and dynamic characterizations of a micromirror unit

**Fig. 4:**
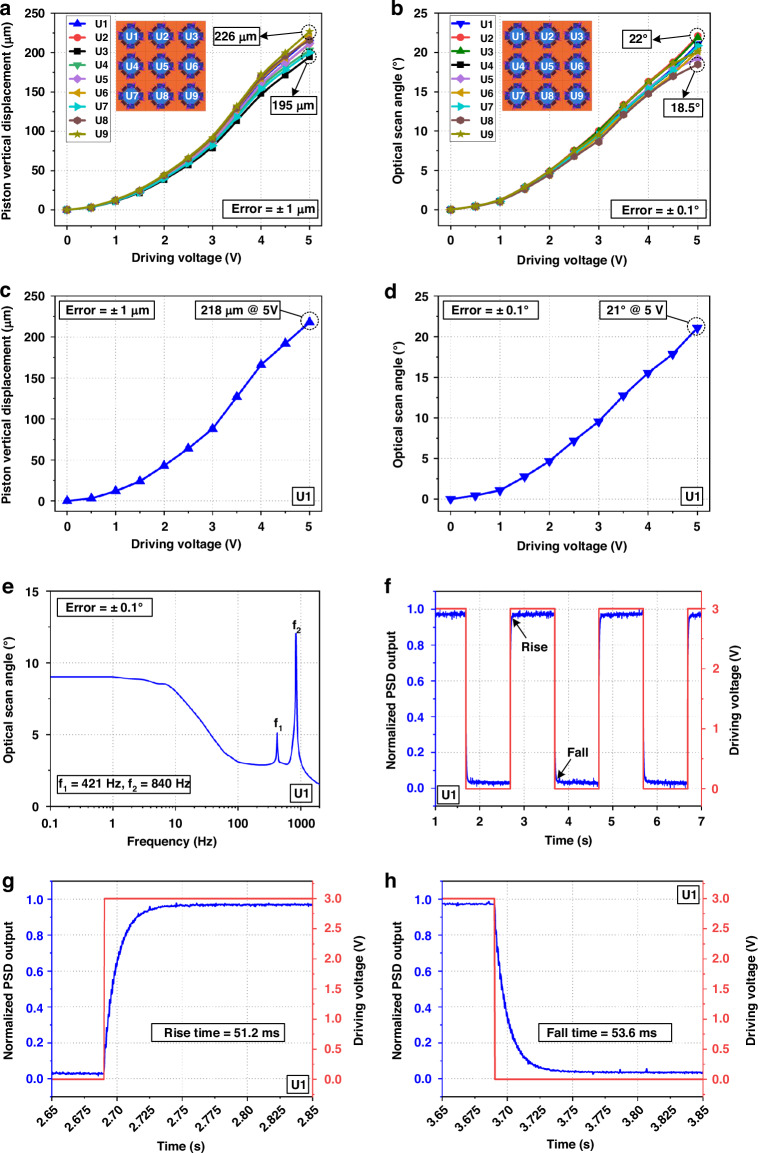
Quasi-static and dynamic characterizations of micromirror units in the MMA. **a** Piston vertical displacement versus driving voltage applied to four actuators. **b** Tip-tilt optical scan angle versus driving voltage applied to one actuator. **c** The piston vertical displacement under different driving voltages in micromirror U1. **d** The optical scan angle under different driving voltages in micromirror U1. **e** Frequency response of micromirror U1. **f** Step response of micromirror U1. **g**, **h** Magnified images of the rise time and the fall time

The quasi-static and dynamic performance of different micromirror units (U1-U9) of the MMA are shown in Fig. 4. In Fig. 4a, with a 5 Vdc driving voltage applied on all four actuators, the maximum and minimum vertical displacements of the micromirror units are 226 μm and 195 μm, respectively. In Fig. 4b, with a 5 Vdc driving voltage applied on one of the four actuators, the maximum and minimum optical scan angle of the micromirror units are 22° and 18.5°, respectively. The main reasons for the inconsistencies in the quasi static performance of the MMA can be explained as follows. The variations in the thickness and shape of the Pt film, resulted from the fabrication processes, lead to the resistance variations of the electrothermal multimorph (PSPI/Al/SiO_2_) actuators. Similarly, the thickness variations of the PSPI, Al and SiO_2_ films cause responsivity discrepancies among the actuators. These inconsistencies in resistance and responsivity discrepancies become more pronounced with the increase in the driving voltage leading to significant differences in the vertical displacement and the optical scan angle among micromirror units.

The subsequent discussions focus on micromirror U1. Figure 4c and d show the quasi-static characterizations of the micromirror U1, where the piston vertical displacement and tip-tilt optical scan angle of the mirror plate can respectively reach 218 μm and 21° at only 5 V_dc_. The nonlinear piston displacement at the high-power input is mainly caused by the thermal annealing effect due to the high temperature in the heated electrothermal multimorph actuators^31^. The linear range in the middle is the combined result of the quadratic relationship between temperature and voltage and the temperature dependence of the electrical resistance of the heater^32^. This nonlinear driving affects the linearity of the position sensors output, which requires a calibration using optical measurement.

The frequency response is measured by applying a sinusoidal wave voltage ranging from 0 V to 3 V to a LSF electrothermal actuator of the micromirror unit, and the results are shown in Fig. 4c, where two resonant peaks at 421 Hz and 840 Hz are observed in the frequency range of 0.1–2000 Hz. The step response of the micromirror unit is also measured with a square waveform varying between 0 and 3 V applied to the actuator. As shown in Fig. 4d–f, the rise time (10% to 90%) is 51.2 ms and the fall time (90% to 10%) is 53.6 ms for the micromirror U1. The 3 dB cutoff frequency due to thermal response is about 20 Hz. As shown in Fig. 4e, it can be seen that the step response has nearly no overshooting even though the mirror design is under-damped with a Q-factor of approximately 50. This is because of thermal delay^33^. In other words, the response time is independent of the frequency of the square-wave driving signal. Table S5 lists the response times of the micromirror units (U1-U9), in which the maximum and minimum value are 53.3 ms and 45.7 ms, respectively. In the final step of the device fabrication process, the release of the mirror plate is achieved by completely etching away the silicon beneath the multimorph. However, in MMAs, completely removal of the silicon is difficult to achieve and the residual silicon is different for different units due to process variations, which cause different response times. In this work, the response time of the electrothermal LSF multimorph actuator is 51.2 ms, which is smaller than the 68 ms reported in the previous work^30^, indicating a faster response speed. The response time of the LSF actuator depends on the thermal resistance and thermal capacitance of the actuator. The actuator’s smaller thermal capacitance can be achieved by narrowing actuator beams, and smaller thermal resistance can be obtained by adding an Al layer to the actuator’s junction to the substrate for better thermal contact, indicating that a smaller response time can be achieved.

#### Quasi-static and dynamic characterizations of the position sensors

**Fig. 5:**
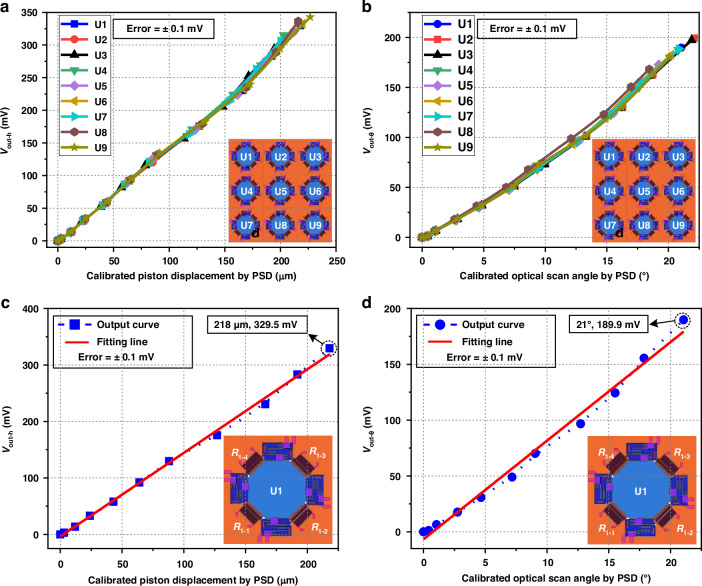
Quasi-static characterizations of the position sensors in micromirror units. **a** Output voltage *V*_out-h_ versus the piston vertical displacement of the mirror plate. **b** Output voltage *V*_out-θ_ versus the tip-tilt optical scan angle of the mirror plate. **c**, **d** Quasi-static characterizations of the position sensors in U1

The quasi-static performance of the position sensors are measured under driving voltage ranging from 0 Vdc to 5 Vdc applied on actuators. The heater of the sensor is activated with a power of 1 mW. The offset of the Wheatstone bridge circuit caused by different initial resistances of thermistors in fabrication can be reduced through the adjustable resistor (RV). Figure 5a, b plots the calibration results of the quasi-static piston sensing and tip-tilt sensing of the position sensors in micromirror U1-U9, respectively. The sensing range of the sensors fully covers the motion range of the mirror plate. The motion range is determined by the quasi-static characteristics of the micromirror. The test results of all position sensors are summarized in Table S6, in which the sensitivities of the position sensors in different micromirror units exhibit an excellent consistency. The discrepancies in the position sensor linearity are mainly attributed to the inherent nonlinearity in the whole scan range. The subsequent discussions focus on micromirror U1. In Figs. 5c, d, the thermistor *R*_1-1_ and *R*_1-2_ are connected to the Wheatstone bridge circuit, which are used to sense the tip-tilt optical scan angle. Thermistor *R*_1-3_ is used to sense the piston displacement. The test results show that the sensing range of the position sensor can cover 218 μm in piston vertical displacement and 21° in tip-tilt optical scan angle. The sensitivities of the piston sensing and tip-tilt sensing are 1.5 mV/μm and 8.8 mV/° with linearities of 3.2% and 5.5%, respectively.

**Fig. 6:**
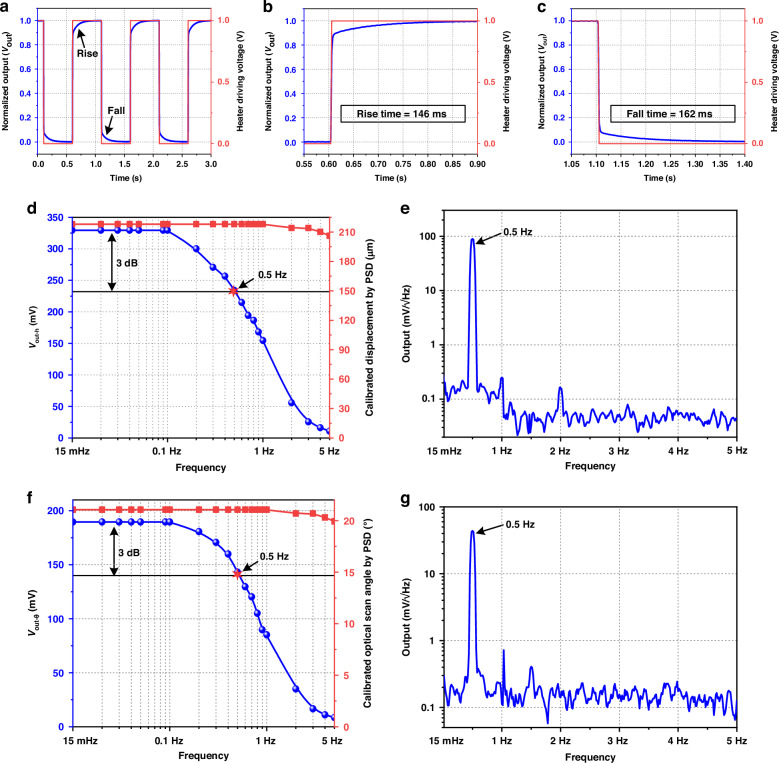
Dynamic characterizations of the position sensor in micromirror U1. **a** Time response. **b** and **c** Magnified images of the rise time and fall time. **d** Frequency response of the position sensor in piston sensing. **e** Output spectrum of piston sensing. **f** Frequency response of the position sensor in tip-tilt sensing. **g** Output spectrum of tip-tilt sensing

The dynamic characterization of the position sensors in micromirror U1 is tested and shown in Fig. 6. The time response of the position sensor is also characterized by applying a square wave varying between 0 V and 1 V to the heater, and the measured results are shown in Figs. 6a–c. It can be seen that the 10% to 90% rise and 90% to 10% fall time are 146 ms and 162 ms, respectively. The frequency response of the sensor in piston vertical displacement is then measured. With the mirror plate approaches the substrate, the calibrated piston vertical displacement by PSD and the measured *V*_out-h_ versus frequency of the driving signal actuating the four actuators are obtained and compared in Fig. 6d, where a sinusoidal driving signal with the amplitude range of 0-5 V is applied to the actuators and a piston sensing range of 218 μm is achieved. Results show that the value of *V*_out-h_ gradually decreases with the increase of the driving signal frequency, and the 3 dB cutoff frequency of the sensor for piston sensing is 0.5 Hz. Figure 6e plots a frequency spectrum (15 mHz to 5 Hz) of piston sensing at 0.5 Hz under a sinusoidal driving signal of 0–5 V on the actuators. Accordingly, the piston sensing resolution is estimated to be approximately 76 nm, which results in a dynamic piston sensing range of 69 dB. This resolution is adequate to satisfy the requirements for OXCs systems.

The frequency response and spectrum of the position sensor for tip-tilt sensing are also measured. Figure 6f plots the calibrated tip-tilt optical scan angle by PSD and the measured *V*_out-θ_ versus the frequency of the applied sinusoidal wave. Results show that the sensor can achieve a tip-tilt sensing range of 21°, and the value of *V*_out-θ_ also gradually decreases with the increase of the driving signal frequency. The 3 dB cutoff frequency of the sensor for tip-tilt sensing is also 0.5 Hz. Figure 6g plots a frequency spectrum of tip-tilt sensing at 0.5 Hz under a sinusoidal driving signal of 0–5 V to one actuator. Similarly, the tip-tilt sensing resolution is estimated to be 0.01°, which also satisfies the requirements of the OXCs systems. The dynamic tip-tilt sensing range is 64 dB. Table S8 lists the comparison of key parameters of the electrothermal MMAs, in which the MMA in this work demonstrates enhanced performance in vertical displacement, optical scanning angle, sensor response time, and sensing range. Note that the key factor affecting the sensitivity of the sensor is the gain of the voltage amplifier following the Wheatstone bridge circuit. In previous work^29^, the gains of the voltage amplifier in piston sensing and tip-tilt sensing are about 500 and 1000, respectively. Since the sensing range in this work is larger than in previous work. The gains of the voltage amplifier in piston sensing and tip-tilt sensing are both reduced to 100, which is to prevent saturation in the large-range sensing. As a result, the sensitivities in this work are lower than that in previous studies.

#### Thermal crosstalk characterization of different position sensors

**Fig. 7:**
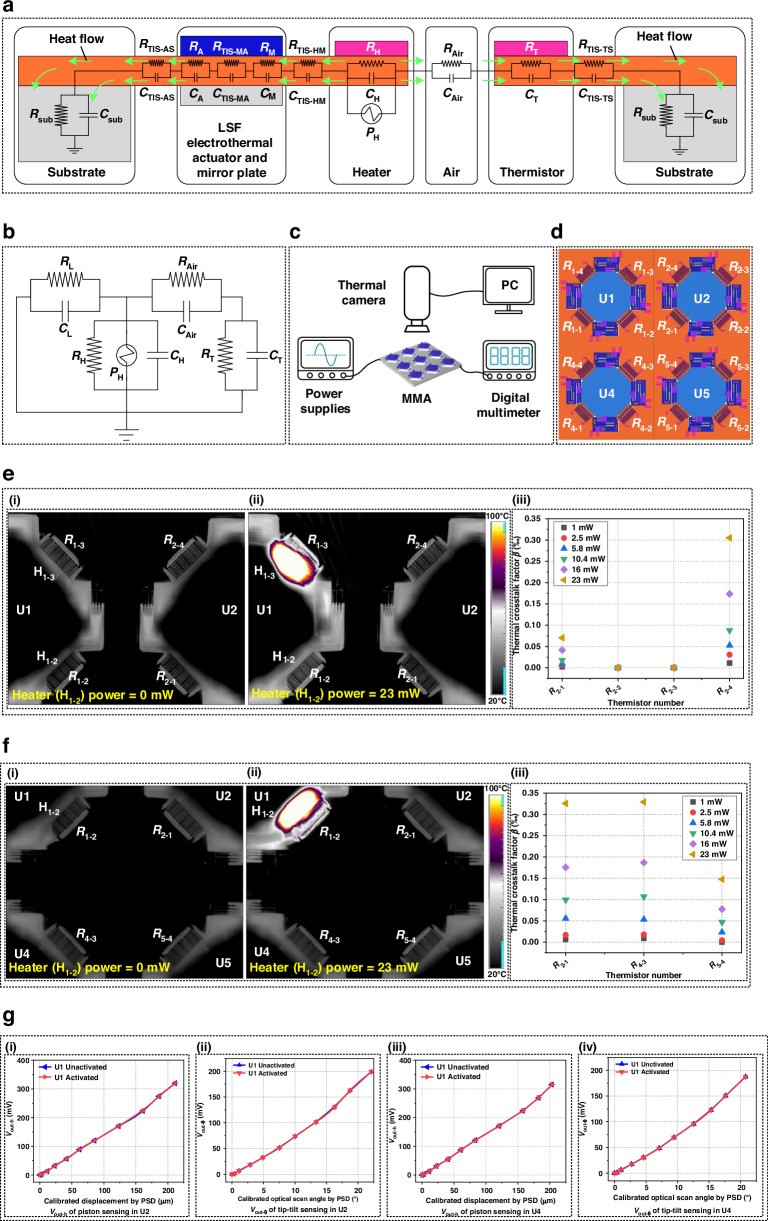
Thermal crosstalk characterizations of different position sensors. **a** Heat transfer model of the sensor. **b** Simplified LEM of the sensor. **c** Test setup for thermal crosstalk characterizations. **d** Illustration of the position relationship of the measured sensors. **e** (i) Initial and (ii) in-operation temperature field distributions when H_1-1_ heats. (iii) The thermal crosstalk in U2 induced by H_1-1_. **f** (i) Initial and (ii) in-operation temperature field distributions when H_1-2_ heats. (iii) The thermal crosstalk in U2, U4 and U5 induced by H_1-2_. **g** The position sensors outputs of U2 and U4 while U1 is activated or unactivated. (i) and (ii) Position sensors outputs of U2. (iii) and (iv) Position sensors outputs of U4

As the surrounding air is heated during the working of the position sensor based on thermal convection, it is necessary to analyze the thermal crosstalk phenomenon. Figure 7a shows the one-dimensional heat transfer model of the position sensor, where the heat generated by the heater can flow to the substrate in two directions including the left one through the mirror plate and actuator, and the right one through air and the thermistor. The heater transfer process in the mirror plate position sensor can be treated as one-dimensional model since the lengths of the heater, actuator, thermistor and substrate are much larger than their thicknesses. The one-dimensional heat transfer equation is given by^34^

1 $$\left(\frac{{{T}}_{{L}}+{{T}}_{{R}}-{2}{{T}}_{{C}}}{{(\Delta {x})}^{2}/(2{kA})}\right)+2{Ag}={2A}\rho {{C}}_{{P}}\frac{{{{d}}}{{T}}_{{C}}}{{{{d}}t}}$$

where *A* is the cross-sectional area perpendicular to the direction of the heat flux, *T*_*L*_, *T*_*R*_ and *T*_*C*_ represent the temperatures at the left boundary, right boundary and center of the heater, respectively, and Δ*x* is the increment in the direction along which the temperature varies, *k*, *g* and *ρC*_*P*_d*T*_*C*_/d*t* denote the thermal conductivity coefficient, the energy generation rate, and the rate of change for the internal energy of the medium. Accordingly, a simplified lumped element model (LEM) of the mirror plate position sensor is shown in Fig. 7b, where *R*_L_ is regarded as the thermal resistance of the heater that accounts for the heat loss through the thermal conduction in the left direction, and the heat loss due to radiation is neglected^34^. *R*_L_ is the series connection of thermal resistance of the thermal isolation structures between the heater and mirror plate *R*_TIS-HM_, thermal resistance of the mirror plate *R*_M_, thermal resistance of the thermal isolation structures between the mirror plate and actuator *R*_TIS-MA_, thermal resistance of the actuator *R*_A_, thermal resistance of the thermal isolation structures between the actuator and substrate *R*_TIS-AS_ and thermal resistance of the substrate *R*_sub_. *C*_L_ is the series connection of thermal capacitance of the thermal isolation structures between the heater and mirror plate *C*_TIS-HM_, thermal capacitance of the mirror plate *C*_M_, thermal capacitance of the thermal isolation structures between the mirror plate and actuator *C*_TSI-MA_, thermal capacitance of the actuator *C*_A_, thermal capacitance of the thermal isolation structures between the actuator *C*_TSI-AS_ and thermal capacitance of the substrate *C*_sub_.

The thermal resistance *R*_L_ and thermal capacitance *C*_L_ of heat loss can be expressed as

2 $${{R}}_{\rm{L}}=\frac{{{L}}_{\rm{L}}}{{{k}}_{\rm{L}}{{A}}_{\rm{L}}}$$

3 $${{C}}_{\rm{L}}={\rho }_{\rm{L}}{{V}}_{\rm{L}}{{C}}_{{\rm{T}}-{\rm{L}}}$$

where *L*_L_, *k*_L_, and *A*_L_ represent the equivalent length, thermal conductivity, and cross-sectional area, respectively, *ρ*_L_, *V*_L_, and *C*_T-L_ denote the equivalent density, volume, and specific heat capacity, respectively. Then the thermal convection resistance *R*_Air_ and thermal convection capacitance *C*_Air_ of air can be expressed as

4 $${{R}}_{\rm{Air}}=\frac{1}{{{h}}_{\rm{Air}}{{S}}_{\rm{Air}}+{{k}}_{\rm{Air}}{{S}}_{\rm{Air}}/{{d}}_{{\rm{Air}}}}$$

5 $${{C}}_{\rm{Air}}={\rho }_{\rm{Air}}{{V}}_{\rm{Air}}{{C}}_{{\rm{T}}-{\rm{Air}}}$$

where *h*_Air_, *S*_Air_, and *k*_Air_ represent convective heat transfer coefficient of air, the contact area of the heater and air, thermal conductivity of air, respectively. *d*_Air_ is the effective thickness of the air surrounding the heater and can be treated as the distance between the heater and thermistor, *ρ*_Air_, *V*_Air_, and *C*_T-Air_ denote the air density, volume, and specific heat capacity, respectively.

According to Eq. (4), a larger *d*_Air_ leads to a larger *R*_Air_. In Eq. (5), *V*_Air_ is proportional to *d*_Air_, which means that a larger *d*_Air_ also results in a larger *C*_Air_. Due to existence of *R*_L_, the heat flow is quite difficult to flow through the mirror plate and actuator to substrate. Moreover, considering the volumes of the mirror plate, actuator, and substrate are much larger than that of the heater, the limited heater power (*P*_H_) can hardly fully charge the thermal capacitance *C*_L_. However, the *R*_Air_ and *C*_Air_ are both much smaller than *R*_L_ and *C*_L_. Hence, most of the heat flows through the air to the thermistor. The thermal resistance *R*_L_ and capacitance *C*_L_ of heater loss are neglected. As shown in Fig. 7b, the response time *τ* of the sensor can be expressed as

6 $$\tau ={{C}}_{\rm{H}}{{R}}_{\rm{H}}+{{C}}_{\rm{Air}}{{R}}_{\rm{Air}}+{{C}}_{\rm{T}}{{R}}_{\rm{T}}$$

where *R*_H_ is the thermal resistance of the heater itself. *C*_H_ is the thermal capacitance of the heater itself. *R*_T_ and *C*_T_ are the thermal resistance of the thermistor and the thermal capacitance of the thermistor, respectively. According to Eq. (1), the smaller *C*_H_, *R*_H_, *C*_T_ and *R*_T_ can be achieved by reducing the volume of the heater and the thermistor, which can reduce the response time. The smaller *C*_Air_ and *R*_Air_ can be achieved by narrowing the distance between the heater and the thermistor, which also can reduce the response time. Additionally, in future work, the MMA could be packaged in a closed chamber, with the ambient gas replaced by another gas with a smaller specific heat capacity and larger thermal conductivity than air.

Furthermore, the thermal crosstalk among different position sensors is characterized when specific sensors are in operation, and the test setup is shown in Fig. 7c, where a thermal camera is used to capture the temperature field distributions of the sensors, and the thermal crosstalk is reflected by the resistance change of the thermistors in different micromirror units. Assuming *R*_0_ is the initial resistance value of the thermistor and Δ*R* represents the resistance change caused by the heater of the adjacent position sensor. The thermal crosstalk factor *β* can be expressed as

7 $$\beta =\frac{\Delta {R}}{{{R}}_{0}}$$

The temperature distributions of the heater H_1-1_ in micromirror unit U1 under activated and unactivated states are shown in Fig. 7e(i) and 7e(ii), respectively. In Fig. 7e(ii), the temperature at the thermistor *R*_1-4_ increases significantly, while the temperature variations at thermistors *R*_2-4_ and *R*_2-1_ in U2 are minimal. Figure 7e(iii) shows the values of thermal crosstalk factor *β* for thermistors in U2 under different heater powers. The thermal crosstalk factors for *R*_2-2_ and *R*_2-3_ in U2 are nearly zero, indicating no change in resistance. This is attributed to their large distance from H_1-1_, making them unaffected by thermal crosstalk. However, the thermal crosstalk factors for *R*_2-1_ and *R*_2-4_ increase with the heater power. Since *R*_2-4_ is closest to the heater, its thermal crosstalk factor is the highest at the same heater power, implying a greater influence from thermal crosstalk. This observation result also corroborates the theoretical analysis. Due to large pitch *d* (3 mm) between micromirror units, the thermal crosstalk factor *β* for *R*_2-4_ is only 0.32‰ even at the heater power of 23 mW. Thus, the thermal crosstalk between adjacent micromirror units is minimal. To further investigate the thermal crosstalk in the MMA. Figure 7f(i) and 7f(ii) show the thermal distribution in U2, U4, and U5 when heater H_1-2_ in U1 is activated. Figure 7f(iii) shows the variations in thermal crosstalk factors at the thermistors. Since *R*_5-4_ is furthest from H_1-2_ it has the smallest thermal crosstalk factor, which means the least influence from thermal crosstalk.

To verify whether the sensor output is affected by thermal crosstalk, the piston sensing and tip-tilt sensing of position sensors in adjacent U2 and U4 are tested with U1 activated. The heater power in all position sensors was maintained at 1 mW. Figure 7g(i) and 7g(ii) show the outputs of the position sensor in U2. It can be seen that the output voltage and sensing range of the position sensor in U2 are not affected by U1. Similarly, Fig. 7g(iii) and 7g(iv) show that the U4 position sensor is also unaffected by U1. This result further confirms that the thermal crosstalk among different sensors in this micromirror array is minimal.

## Discussion

The most pronounced advantage of tip-tilt-piston (TTP) electrothermal micromirror arrays (MMAs)^10,11^, compared to other types of MMAs^12–15^, is their large quasi-static large piston and tip-tilt scan range at low driving voltage. The detailed comparisons are listed in Table S7. Based on its unique superiorities, the low-voltage-driven electrothermal micromirror with a large scanning range is favorable in applications such as optical coherence tomography (OCT), FTS, OXC, and OPA systems. In addition to angular scanning, i.e., tip and/or tilt, micromirrors for FTS and OPA must have piston actuation for phase control.

The unique feature of the MMA reported in this work compared to the previous electrothermal MMAs^10,11^ is the integration of the thermal convection-based mirror plate position sensor. Although there is an attempt to integrate such position sensors in an electrothermal MMA^29^, that MMA has a lower fill-factor (12%), piston scanning range (180 μm), and tip-tilt scanning range (11.8°). Hence, an enhanced TTP electrothermal MMA with integrated position sensors is presented in this work. Table S8 lists the key parameters of the inductive eddy current sensing and optical sensing currently used in single electrothermal micromirror compared the method presented in this work. The sensing range of the position sensor in this work can fully cover the motion range of the MMA. Compared to inductive eddy current sensing^23^, the sensing method proposed in this study offers significant advantages in piston displacement detection resolution (72 nm) and the tip-tilt sensing range (0–21°). Additionally, in comparison to optical sensing^26^, our approach has larger sensing ranges for piston sensing (0–218 μm) and tip-tilt sensing. The parameters of the position sensor in this work are adequate to satisfy the requirements of FTS and OXC systems. Furthermore, compared to the highly challenging application of inductive eddy current sensing and optical sensing in electrothermal MMAs, the fabrication process of the position sensor can completely compatible with the fabrication of the electrothermal MMA, which means the sensor does not need any extra cost.

## Conclusion

We introduced a novel tip-tilt-piston electrothermal MMA containing temperature field-based position sensors. This design is an on-chip integration solution and does not need to assemble off-chip components. A FEM model is built and analyzed to study the behaviors of the micromirror and the sensor. Quasi-static and dynamic characterizations of the micromirror show that its vertical displacement and optical scan angle can reach 218 μm and 21°, respectively. The frequency response of the micromirror is also characterized, where the two resonance frequencies are 421 Hz and 840 Hz, respectively. Besides, the rise time is 51.2 ms and fall time is 53.6 ms. Results show that the sensing range of the position sensor can cover the entire motion of the mirror plate and the sensitivities of piston sensing and tip-tilt sensing are 1.5 mV/μm and 8.8 mV/°, with linearities of 3.2% F.S and 5.5% F.S, respectively. The displacement resolution of 72 nm and optical scan angle resolution of 0.01° are achieved. Furthermore, an LEM based on the heat transfer model of the position sensor is developed and predicts the responsivity and thermal crosstalk of the position sensor, which shows a good agreement with test results. Future effort will be focused on increasing the sensitivity and response speed of the position sensors by optimizing the designs of the heaters and the thermistors. Meanwhile, the mirror plate position sensors will be used in the closed-loop control of electrothermal MMAs to achieve real-time position control of every micromirror unit.

## Supplementary information

Supplemental Material
