# Supplementary material for: A tip-tilt-piston electrothermal micromirror array with integrated position sensors
Source: Microsyst Nanoeng. 2025 Mar 7;11:45. doi: 10.1038/s41378-024-00835-w (PMC11889227; doi:10.1038/s41378-024-00835-w)
Supplement: Supplementary file 1 — Supplemental Material [file 41378_2024_835_MOESM1_ESM.docx]

**Supplementary information**

# A Tip-tilt-piston Electrothermal Micromirror Array with Integrated Position Sensors

**Anrun Ren^1,2^, Yingtao Ding^1,2^, Hengzhang Yang^1,2^, Qiangqiang Liu^1,2^, Teng Pan^1,2^, Ziyue Zhang^1,3^, Huikai Xie^1,2,3 *^**

1. School of Integrated Circuits and Electronics, Beijing Institute of Technology, Beijing 100081, China

2. Engineering Research Center of Integrated Acousto-opto-electronic Microsystems, Ministry of Education of China, Beijing 100081, China

3. Chongqing Institute of Microelectronics and Microsystems, Beijing Institute of Technology, Chongqing 400030, China

*hk.xie@ieee.org

**Supplementary Materials:**

Figures S1-S6

Table S1-S8.

References 1-36.

## Figures


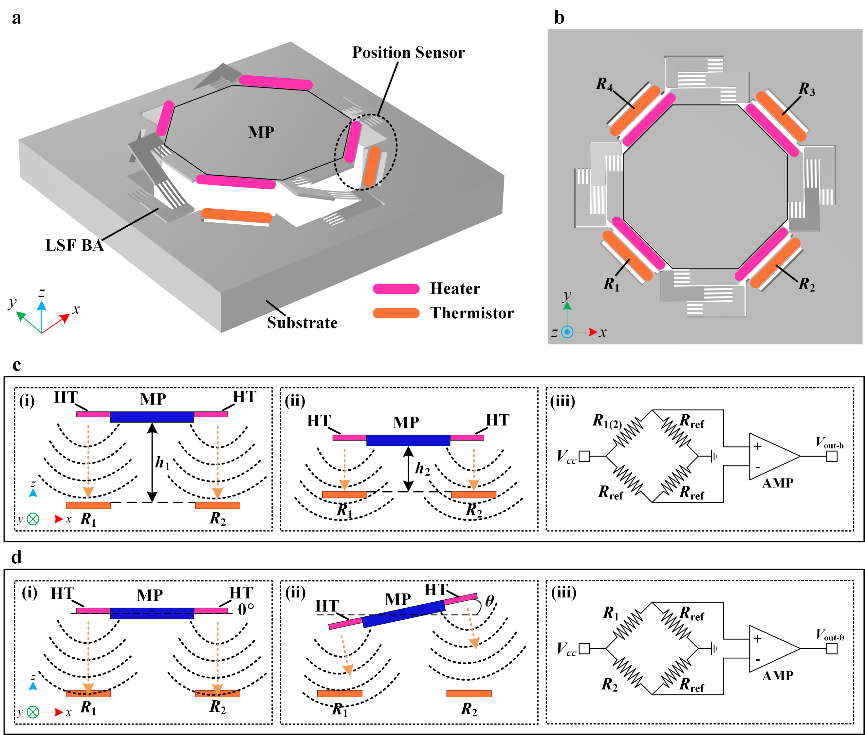


**Figure S1. Schematic illustrations and working principle of position sensors. a** Side view of the micromirror. **b** Top view of the micromirror. **c** Sensing principle of the vertical displacement of the mirror plate. **d** Sensing principle of the tip-tilt angle of the mirror plate. HT: heater. MP: mirror plater. *R*_ref_: Reference resistance used for balancing Wheatstone bridges

Figure S1a and b illustrate the proposed electrothermal micromirror integrated with temperature field-based mirror position sensors, where the central mirror plate is supported by four lateral shift free bimorph actuators (LSF BA), and four temperature field-based mirror plate position sensors are located at the four corners of the mirror plate. Each mirror plate position sensor consists of a heater on the mirror plate and a thermistor on the substrate, where the heater can move with the mirror plate. The working principle of the mirror plate position sensor is illustrated in Figure S1c and d. Once the heaters work, a temperature field is generated nearby, in which the temperature distribution around the thermistors changes with the vertical displacement or tip-tilting angle of the mirror plate. As shown in Figure S1c(i) and (ii), when the mirror plate displaces to different heights (*h*_1_, *h*_2_), the temperature of the thermistors *R*_1_ and *R*_2_ varies. Specifically, the closer to the heater, the higher temperature of the thermistor is, causing the larger resistance change of the thermistor. The resistance change of the thermistor can be further converted to the voltage output by a Wheatstone bridge circuit, as illustrated in Figure S1c(iii), where *R*_ref_ is the reference resistor for balancing the circuit. Accordingly, the vertical displacement of the mirror plate is detected. Similarly, the tip-tilt angle (*θ*) of the mirror plate can also be recognized through *R*_1_ and *R*_2_, as shown in Figure S1d(i) and (ii). When the mirror plate tilts at an angle of *θ* as indicated in Figure S1d(ii), *R*_1_ is closer to the heater than *R*_2_, thus its resistance change is larger. The resistance changes are converted by the Wheat bridge circuit in Figure S1d(iii) and the *θ* can be detected. The total temperature changes can be calculated as follows:

Δ*Τ*_h_ = *Τ*(*R*_1_) – *T*(*R*_ref_) (1)

Δ*Τ*_θ_ = *Τ*(*R*_1_) – *T*(*R*_2_) (2)

where Δ*Τ*_h_ denotes the temperature difference induced by the vertical displacement change of the mirror plate, Δ*Τ*_θ_ denotes the temperature difference induced by the mirror plate tilting, *T*(*R*_i_) is the temperature of the thermistor *R*_i_, i represents one of the four thermistors, and *R*_ref_ is the reference resistor for balancing the Wheatstone bridge circuit. The output voltage of the Wheat bridge circuit can be expressed as follows:

*V*_out-h_ = A*α*Δ*T*_h_ · *V_cc_*  (3)

*V*_out-θ_ = A*α*Δ*T*_θ_ · *V_cc_*  (4)

where A, *α* and *V*_cc_ are the constant of the circuit, the temperature coefficient of resistance (TCR) of the thermistor and the supply voltage of the circuit, respectively.


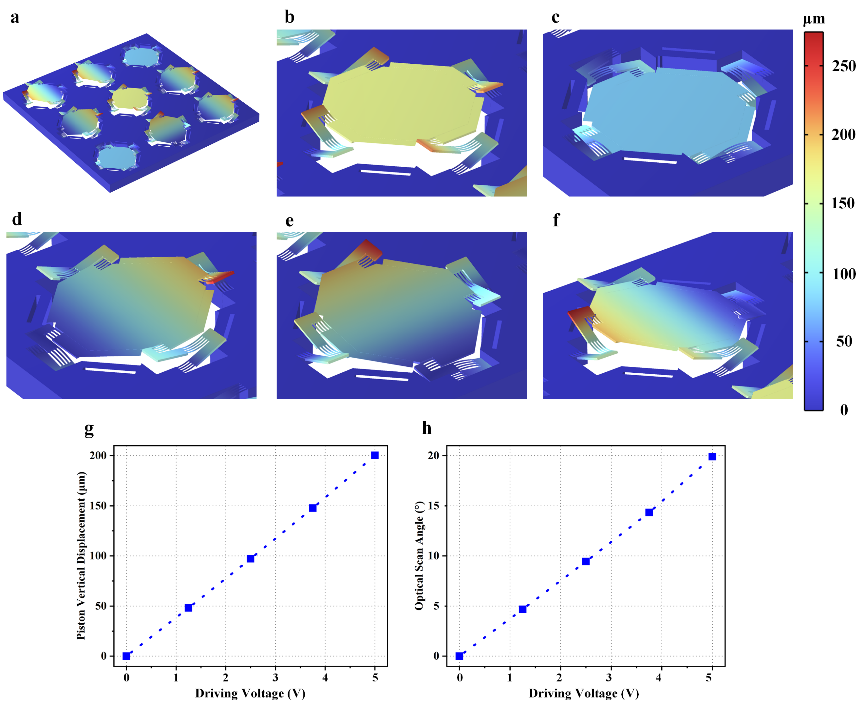
 **Figure S2.** Simulation results of the MMA. **a** Different motions of the micromirror units in the MMA. **b** Initial position. **c** Piston motion by exciting the four actuators. **d,** **e and f** Tip-tilt motion by exciting only one actuator. **g** and **h** Piston vertical displacement and tip-tilt optical angle versus driving voltage of actuators.


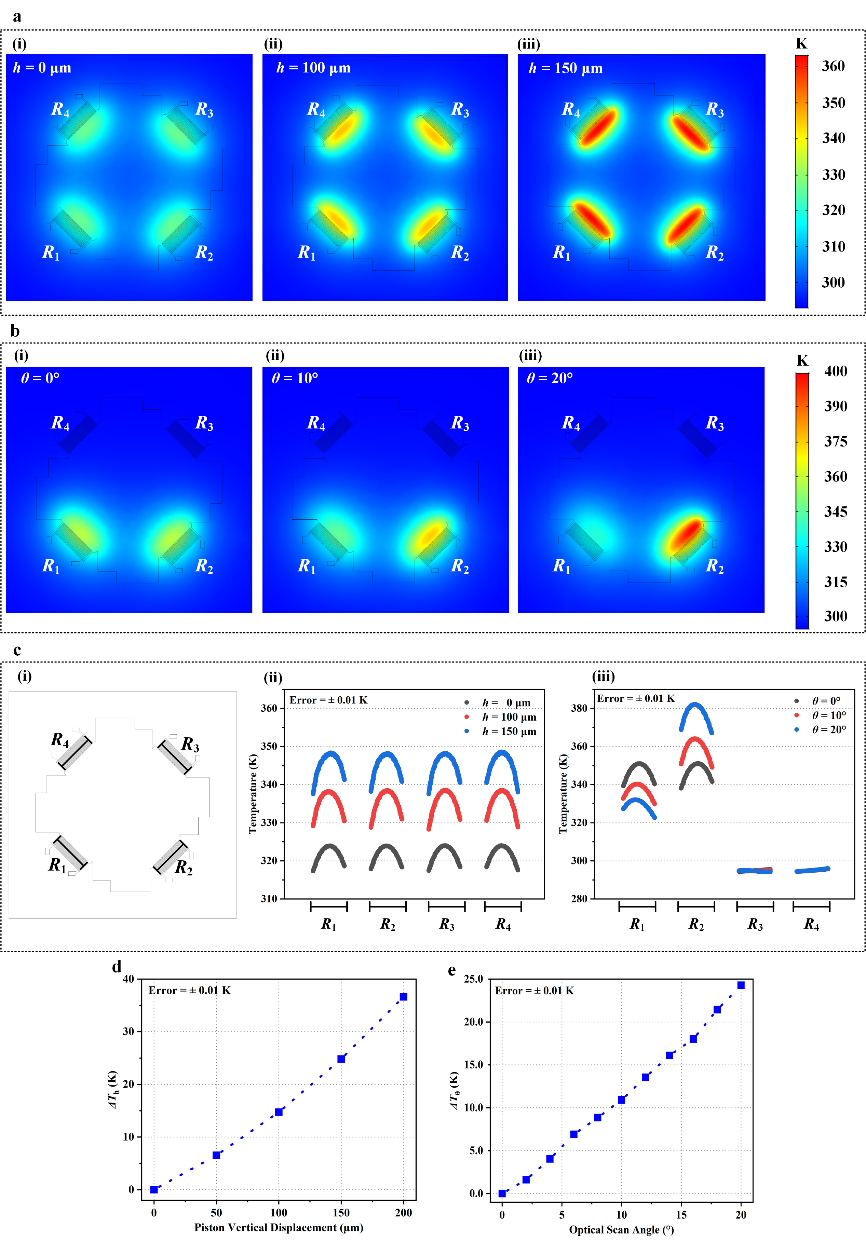


**Figure S3. Simulation results of the position sensors. a** and **b** Temperature field distributions near the thermistors for different piston vertical displacements and tip-tilt optical scan angle. **c** Temperature value curves along the thermistors in **a** and **b**. **d** and **e** Temperature variation curves for piston sensing and tip-tilt sensing.


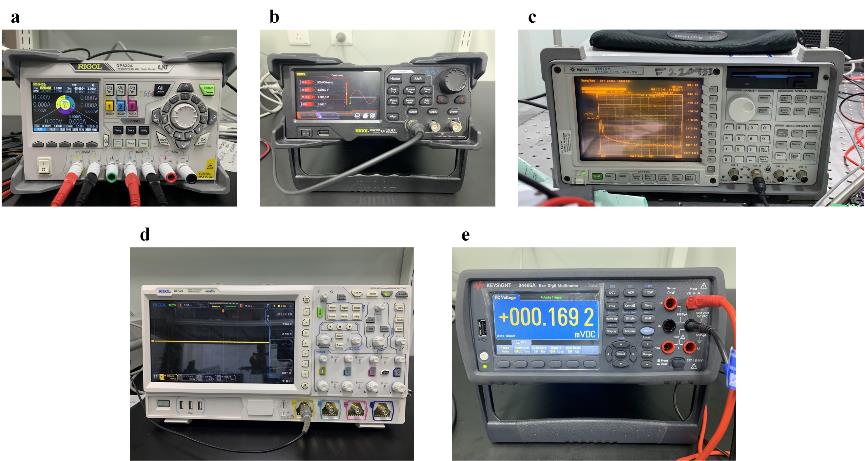


**Figure S4. Power supplies and testing instruments. a** DC power supply. **b** Functional waveform generator. **c** Spectrum analyzer. **d** Oscilloscope. **e** Digital multimeter.


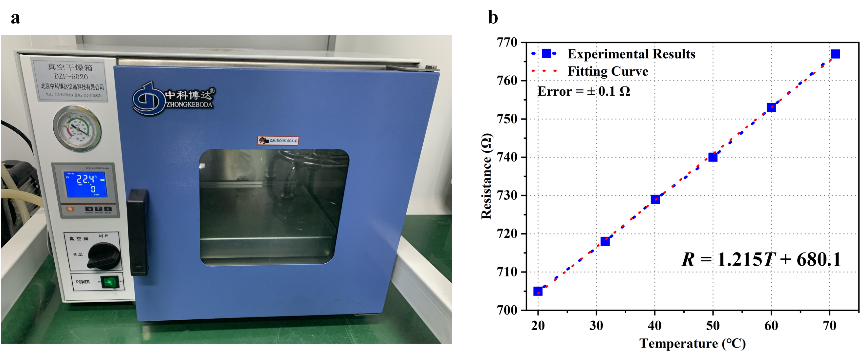


**Figure S5. Measurement of the TCR of the Pt resistance. a** Temperature-controlled oven. **b** Resistance-temperature curve. The TCR of the resistance can be calculated as the ratio of the slope to the Resistance-axis intercept[35].


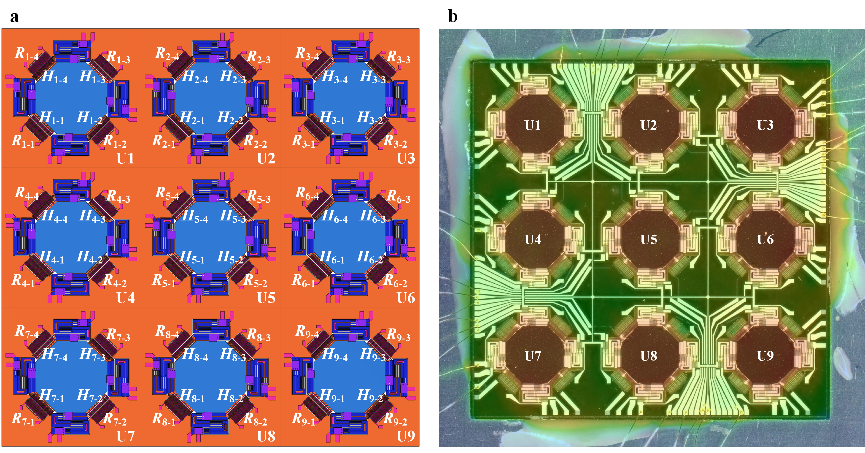


**Figure S6. The MMA device. a** The numbers of position sensors in each micromirror unit (U1-U9). **b** A top view of optical photo after package. Hi and *R*i denote the heater and thermistor, respectively. i represents one of the four heaters and thermistors.

## Tables

Table S1. Properties of the employed materials[36]

| Material | Young’s modulus (GPa) | CTE (ppm·K^-1^) | Thermal conductivity (W·m^-1^·K^-1^) | Electrical conductivity (S·m^-1^) |
| --- | --- | --- | --- | --- |
| Al | 70 | 23.1 | 237 | 35.5 × 10^6^ |
| SiO_2_ | 70 | 0.5 | 1.4 | Insulator |
| Pt | 168 | 8.8 | 71.6 | 8.9 × 10^6^ |
| PSPI | 2.3 | 20 | 0.15 | Insulator |

Table S2. Parameters used in the thermal convection simulations[34]

| Parameter | Value |
| --- | --- |
| Initial temperature (*T*_0_) | 293.15 K |
| Initial pressure (*p*_0_) | 101.325 kPa |
| Air thermal conductivity (*k*_air_) | 0.0267 W/(m·K) |
| Air viscosity coefficient (*μ*_air_) | 2.3 |
| Air specific heat capacity (*c_p_*_,air_ ) | 1.004 kJ/(kg·K) |
| Universal gas constant (*R_gas_*) | 8.314 J/(mol·K) |
| Convective heat transfer coefficient (*h*) | 1900 W/(m^2^·K) |

Table S3. Specific equipment name and model

| Equipment Name | Type |
| --- | --- |
| DC power supply | RIGOL DP832A |
| Functional waveform generator | RIGOL DG2102 |
| Spectrum analyzer | AGILENT 35670A |
| Oscilloscope | RIGOL DS7024 |
| Digital multimeter | KEYSIGHT 34465A |

Table S4. Resistance values of heaters and thermistors in the position sensors (Ω)

|  | U1 | U2 | U3 | U4 | U5 | U6 | U7 | U8 | U9 |
| --- | --- | --- | --- | --- | --- | --- | --- | --- | --- |
| *H*_1_ | 1541.65 | 1576.78 | 1510.817 | 1576.67 | 1572.01 | 1550.23 | 1551.3 | 1586.66 | 1581.881 |
| *H*_2_ | 1521.33 | 1587.90 | 1500.77 | 1520.82 | 1490.04 | 1479.59 | 1504.75 | 1597.85 | 1530.32 |
| *H*_3_ | 1525.36 | 1556.01 | 1475.91 | 1566.96 | 1571.20 | 1566.81 | 1494.85 | 1504.78 | 1534.29 |
| *H*_4_ | 1551.32 | 1495.40 | 1520.29 | 1543.16 | 1560.15 | 1527.41 | 1514.01 | 1546.17 | 1518.08 |
| *R*_1_ | 5391.81 | 5283.97 | 5315.69 | 5479.37 | 5318.67 | 5418.41 | 5500.21 | 5514.66 | 5553.53 |
| *R*_2_ | 5331.43 | 5259.40 | 5366.07 | 5224.14 | 5305.84 | 5303.68 | 5436.46 | 5446.41 | 5234.36 |
| *R*_3_ | 5411.86 | 5501.43 | 5249.51 | 5462.96 | 5338.46 | 5230.06 | 5491.37 | 5452.93 | 5171.48 |
| *R*_4_ | 5341.19 | 5535.19 | 5462.92 | 5180.54 | 5386.03 | 5574.58 | 5498.03 | 5269.03 | 5518.47 |

Table S5 Quasi-static and dynamic performances of the micromirror units in the MMA

| Micromirror units | U1 | U2 | U3 | U4 | U5 | U6 | U7 | U8 | U9 |
| --- | --- | --- | --- | --- | --- | --- | --- | --- | --- |
| Piston scan range (μm) | 218 | 211 | 195 | 203 | 212 | 220 | 200 | 216 | 226 |
| Tip-tilt scan range (°) | 21 | 22 | 21.9 | 20.8 | 19.2 | 20.2 | 20.9 | 18.5 | 20.1 |
| Response time (ms) | 51.2 | 49.7 | 45.7 | 48.6 | 49.8 | 51.6 | 47.5 | 52.3 | 53.3 |
| Fall time (ms) | 53.6 | 51.8 | 48.3 | 51.4 | 52.1 | 53.9 | 51.6 | 56.2 | 55.8 |
| f1 (Hz) | 421 | 436 | 427 | 429 | 417 | 433 | 418 | 405 | 423 |
| f2 (Hz) | 840 | 881 | 850 | 871 | 825 | 873 | 846 | 817 | 855 |

Table S6 Quasi-static characterizations of the position sensors in all micromirror units

| Units | Piston sensing | | | Tip-tilt sensing | | |
| --- | --- | --- | --- | --- | --- | --- |
|  | Sensitivities | Sensing range | Linearities | Sensitivities | Sensing range | Linearities |
| U1 | 1.5 mV/μm | 218 μm | 3.2% | 8.8 mV/° | 21° | 5.5% |
| U2 | 1.5 mV/μm | 211 μm | 1.8% | 8.8 mV/° | 22° | 6.1% |
| U3 | 1.5 mV/μm | 195 μm | 0.8% | 8.8 mV/° | 21.9° | 5.9% |
| U4 | 1.5 mV/μm | 203 μm | 3.9% | 8.8 mV/° | 20.8° | 6.0% |
| U5 | 1.5 mV/μm | 212 μm | 1.8% | 8.8 mV/° | 19.2° | 5.8% |
| U6 | 1.5 mV/μm | 220 μm | 1.8% | 8.8 mV/° | 20.2° | 4.5% |
| U7 | 1.5 mV/μm | 200 μm | 3.0% | 8.8 mV/° | 20.9° | 6.0% |
| U8 | 1.5 mV/μm | 216 μm | 4.9% | 8.9 mV/° | 18.5° | 6.1% |
| U9 | 1.5 mV/μm | 226 μm | 2.2% | 8.8 mV/° | 20.1° | 5.6% |

Table S7 Comparison of key parameters of the MMAs

| Refs. | Driving method | DoFs | Driving voltage | Piston displacement | Tip-tilt optical scan angle | Mirror array number |
| --- | --- | --- | --- | --- | --- | --- |
| Wu *et al.* [10] | Electrothermal | TTP | 4.5 V | 200 μm | 18° | 4 × 4 |
| Jia *et al.* [11] | Electrothermal | TTP | 8 V | 310 μm | 21° | 4 × 4 |
| Ji *et al.* [13] | Electromagnetic | TT | 21 V | - | 90° | 4 × 4 |
| Jung *et al.* [14] | Electrostatic | TTP | 160 V | 70 nm | 1.8° | 5 × 5 |
| Bruno *et al.* [15] | Piezoelectric | TT | 200 V | - | 0.85° | 2 × 2 |

Table S8 Comparison of key parameters of different sensing methods applied on electrothermal micromirrors

|  | Piston sensing range | Piston sensing resolution | Piston sensitivities | Tip-tilt sensing range | Tip-tilt sensing resolution | Tip-tilt sensitivities | Response times |
| --- | --- | --- | --- | --- | --- | --- | --- |
| Inductive eddy current sensing [23] | 500 μm | 96 nm | 4.15 kHz/μm | 1.06° | 0.0013° | 60.5 kHz/° | - |
| Optical sensing [26] | 190 μm | - | - | 5° | 0.0067° | - | - |
| Previous work [29] | 180 μm | - | 8.6 mV/μm | 11.8 | - | 95.5 mV/° | 158 ms |
| This work | 218 μm | 72 nm | 1.5 mV/μm | 21° | 0.01° | 8.8 mV/° | 146 ms |

## References

1. Premalatha, S. et al. Machine learning aided design and optimization of MEMS optical phased array with silicon micro mirrors for nanofabrication. *Opt. Quantum Electron.* **55**, 13 (2023).

2. Wang, Y. et al. 2D broadband beamsteering with large-scale MEMS optical phased array. *Optica*. **16**, 557-562 (2019).

3.Hamann, S. et al. High-speed random access optical scanning using a linear MEMS phased array. *Opt. Lett.* **43**, 5455-5458 (2018).

4. Hoskinson, R., Hampl, S. & Stoeber, B. Arrays of large-area, tip/tilt micromirrors for use in a high-contrast projector. *Sens. Actuators A: phys.* **173**, 172-179 (2012).

5. Nakai, A. et al. Double-sided scanning micromirror array for autostereoscopic display. *Sens. Actuators A: phys.* **135**, 80-85 (2007).

6. Jason, B. S. et al. Design and development of a 331-segment tip–tilt–piston mirror array for space-based adaptive optics. *Sens. Actuators A: phys.* **138**, 230-238 (2007).

7. Xiao, X., Dong, X. & Yu, Y. MEMS-based linear micromirror array with a high filling factor for spatial light modulation. *Opt. Express*. **29**, 33785-33794 (2021).

8. Ulrike, D. et al. Analog Spatial Light Modulators Based on Micromirror Arrays. *Micromachines*. **12**, 483 (2021).

9. Chen, K. et al. Modular optical cross-connects (OXCs) for large-scale optical networks, *IEEE Photon. Technol. Lett.* **31**, 763-766 (2019).

10. Wu, L. et al. A tip-tilt-piston micromirror array for optical phased array applications. *J. Microelectromech. Syst.* **19**, 1450-1461 (2010).

11. Jia, K., Samuelson, S. R. & Xie, H. High-fill-factor micromirror array with hidden bimorph actuators and tip–tilt-piston capability. *J. Microelectromech. Syst.* **20**, 573-582 (2011).

12. Jang, Y. & Kim, Y. Design, fabrication and characterization of an electromagnetically actuated addressable out-of-plane micromirror array for vertical optical source applications. *J. Micromech. Microeng*. **13**, 853-863 (2003).

13. Ji, C. & Kim, Y. Electromagnetic micromirror array with single-crystal silicon mirror plate and aluminum spring. *J. Lightwave Technol.* **21**, 584-590 (2003).

14. Jung, I. et al. High Fill-Factor Two-Axis Gimbaled Tip-Tilt-Piston Micromirror Array Actuated by Self-Aligned Vertical Electrostatic Combdrives. *J. Microelectromech. Syst.* **15**, 563-571 (2006).

15. Bruno, B. et al. Micro Fresnel mirror array with individual mirror control. *Smart Mater. Struct.* **29**, 075003 (2020).

16. Zhang, R. et al. An integrated capacitive sensing method for electrostatic comb-drive micromirrors. *Sens. Actuators A: phys.* **357**, 114416 (2023).

17. Lee, M. et al. Capacitive sensing for 2-D electrostatic MEMS scanner in a clinical endomicroscope. *IEEE Sens. J.* **22**, 24493-24503 (2022).

18. Xia, C. et al. A time division capacitive feedback method of electrostatic MEMS mirror driven by PWM signal. *Sens. Actuators A: phys.* **322**, 112631 (2021).

19. Frigerio, P. et al. Piezoresistive versus piezoelectric position sensing in MEMS micromirrors: a noise and temperature drift comparison. *IEEE Sens. Lett.* **6**, 1-4 (2022).

20. Frigerio, P. et al. Long-Term Characterization of a New Wide-Angle Micromirror With PZT Actuation and PZR Sensing. *J. Microelectromech. Syst.* **30**, 281-289 (2021).

21. Aonuma, T. et al. Characteristics and improved design of piezoresistive rotation angle sensor integrated in micromirror device. *Jpn. Appl. Phys.* **48**, 04C191 (2009).

22. Coskun, M. B.et al. Nanoscale displacement sensing using microfabricated variable-inductance planar coils. *Appl. Phys. Lett.* **103**, 143501. (2013)

23. Tseng, V. F. & Xie, H. Simultaneous piston position and tilt angle sensing for large vertical displacement micromirrors by frequency detection inductive sensing. *Appl. Phys. Lett.* **107**, 214102 (2015).

24. Tseng, V. F. & Xie, H. Resonant Inductive Coupling-Based Piston Position Sensing Mechanism for Large Vertical Displacement Micromirrors. *J. Microelectromech. Syst.* **25**, 207-216 (2016).

25. Ishikawa, I. et al. Integrated micro-displacement sensor that measures tilting angle and linear movement of an external mirror. *Sens. Actuators A: phys.* **138**, 269-275 (2007).

26. Liu, Y. et al. Integrated tilt angle sensing for large displacement scanning MEMS mirrors. *Opt. Express.* **18**, 12065-12075 (2010).

27. Ghazinouri, B., He, S., & Tai, T. S. A position sensing method for 2d scanning mirrors, *J. Micromech. Microeng*. **32**, 045007 (2022).

28. Ren, A. et al. Integrated Thermal Convection-Based Position Sensing for Electrothermal Micromirrors. in *2023 22st International Conference on Solid-State Sensors, Actuators and Microsystems*. (IEEE, 2023).

29. Ren, A. et al. An Electrothermal Micromirror Array Integrated with Thermal Convection-Based Mirror Position Sensors. in *2024 IEEE 37th International Conference on Micro Electro Mechanical Systems.* (IEEE, 2024).

30. Yang, H. et al. A robust lateral shift free (LSF) electrothermal micromirror with flexible multimorph beams. *Microsyst. Nanoeng.* **9**, 108 (2023).

31. Pal, S. & Xie, H. Repeatability study of an electrothermally actuated micromirror. in *2009 IEEE International Reliability Physics Symposium* 549–556 (IEEE, 2009).

32. Jia, K., Pal, S. & Xie, H. An electrothermal tip–tilt–piston micromirror based on folded dual S-shaped bimorphs. *J. Microelectromech. Syst.* **18**,1004–1015 (2009).

33. Li, M., Chen, Q., Liu, Y., Ding, Y. & Xie, H. Modeling and experimental verification of step response overshoot removal in electrothermally actuated MEMS mirrors. *Micromachines* **8**, 289–300 (2017).

34. Zhou, L. et al. Investigation of dynamic thermal behaviors of an electrothermal micromirror. *Sens. Actuators A: phys.* **263**, 269-275 (2017).

35. Xiao, L. et al. Analog-controlled light microshutters based on electrothermal actuation for smart windows. *Opt Express*. **28**, 33106 (2020).

36. Pal, S. & Xie, H. Fabrication of robust electrothermal MEMS devices using aluminum–tungsten bimorphs and polyimide thermal isolation. *J. Micromech. Microeng.* **22**, 115036 (2012).
